# Global wave number-4 pattern in the southern subtropical sea surface temperature

**DOI:** 10.1038/s41598-020-80492-x

**Published:** 2021-01-08

**Authors:** Balaji Senapati, Mihir K. Dash, Swadhin K. Behera

**Affiliations:** 1 Centre for Oceans, Rivers, Atmosphere and Land Sciences, Indian Institute of Technology Kharagpur, Kharagpur, West Bengal India; 2 Application Laboratory, VAiG, Japan Agency for Marine-Earth Science and Technology, Yokohama, Kanagawa Japan

**Keywords:** Ocean sciences, Ocean sciences

## Abstract

Exploratory analysis using empirical orthogonal function revealed the presence of a stationary zonal wavenumber-4 (W4) pattern in the sea surface temperature (SST) anomaly in the southern subtropics (20°S–55°S). The signal over the Southern subtropics is seasonally phase-locked to the austral summer and persists up to mid-autumn. Thermodynamic coupling of atmosphere and the upper ocean helps in generating the W4 pattern, which later terminates due to the breaking of that coupled feedback. It is found that the presence of anomalous SST due to W4 mode in the surrounding of Australia affects the rainfall over the continent by modulating the local atmospheric circulation. During positive phase of W4 event, the presence of cold SST anomaly over the south-eastern and -western side of Australia creates an anomalous divergence circulation. This favours the moisture transport towards south-eastern Australia, resulting in more rainfall in February. The scenario reverses in case of a negative W4 event. There is also a difference of one month between the occurrence of positive and negative W4 peaks. This asymmetry seems to be responsible for the weak SST signal to the South of Australia. Correlation analysis suggests that the W4 pattern in SST is independent of other natural variabilities such as Southern Annular Mode, and Indian Ocean Dipole as well as a rather weak relationship with El Niño/Southern Oscillation.

## Introduction

Southern subtropics is a bridge between tropics and Antarctic, and always of interest to the climate scientists, meteorologists, and oceanographers for its dynamics and role of an inter-mediator. Variation in sea surface temperature (SST) over the subtropics has the potential to affect the circumpolar winds and currents, mid-latitude storm tracks, tropical-extratropical teleconnection, meridional atmospheric cells, inter-hemispheric thermohaline circulation and, oceanic shallow sub-tropical cells^1^. In recent decades, the inter-annual variation of SST in the southern subtropics draws attention because of its vital role in the variation of precipitation over the subtropical continents by modulating the regional atmospheric circulation^2–10^. Additionally, weather and climate of the Southern Hemisphere are affected by different modes of climate variations such as El Niño/Southern Oscillation^11^ (ENSO), Indian Ocean Dipole^12^ (IOD), Atlantic Niño^13^, Southern Annular Mode^14^ (SAM), Antarctic Circumpolar Wave^15^, Indian Ocean subtropical dipole^3^ (IOSD), south Atlantic subtropical dipole^10,16^ (SASD) and the South Pacific subtropical dipole^17^ (SPSD).

While these studies focused on ocean–atmosphere coupled modes in the tropics, subtropics, and higher latitudes some other studies revealed presence of stationary waves of wave number-1 and -3 in southern subtropical atmosphere^18^. It is suggested that the wave number-1, is related to the variability of cold lows over the Australia-New Zealand with ridge formation in the sub-tropical eastern Atlantic and sub-polar central Pacific. On the other hand, the wave number-3 is linked to the weather over land areas through the presence of ridges nearer to the subtropical continents. These waves have the potential to affect climate in the global as well as regional scale by modifying the movement of pressure systems in the west-wind belt^19–23^.

Though less known, some studies have reported the presence of global wave number-4 (W4) pattern in the Southern subtropical atmosphere^16,24,25^. Additionally, a W4 pattern had already been observed in the average brightness chart of 1969 over the subtropical latitude^24^. The behaviour of atmospheric variables seems to follow a W4 pattern during the co-variability of the Indian and Atlantic Ocean subtropical dipoles^16^ and was responsible for South Africa floods in January, 2013^25^. At the same time, SST response to the atmosphere is found to be of a wavenumber-3 type in a global view^16,25,26^. Recently, it is reported that tropical Rossby waves have the potential to generate a zonal W4 pattern in the southern Indo-Atlantic Ocean basin that affects precipitation over Australia^27^.

From the past studies, the question arises whether W4 pattern is evident only in the atmospheric circulations as waves 1–3^18^, or it is extended to the ocean like that of zonal wave number-3^26^. To explore such a relationship and to extend the work of Yasunari^24^, for the first time this study included the Pacific Ocean along with the Indo-Atlantic sector to analyze the inter-annual variation of SST over the southern subtropics on a global scale. Together with the generation mechanism, presence of an interesting global W4 mode in the subtropical SST and its impact on south-eastern Australian rainfall are discussed.

## Results

### Wave number-4 pattern in the Southern subtropical SST

**Figure 1:**
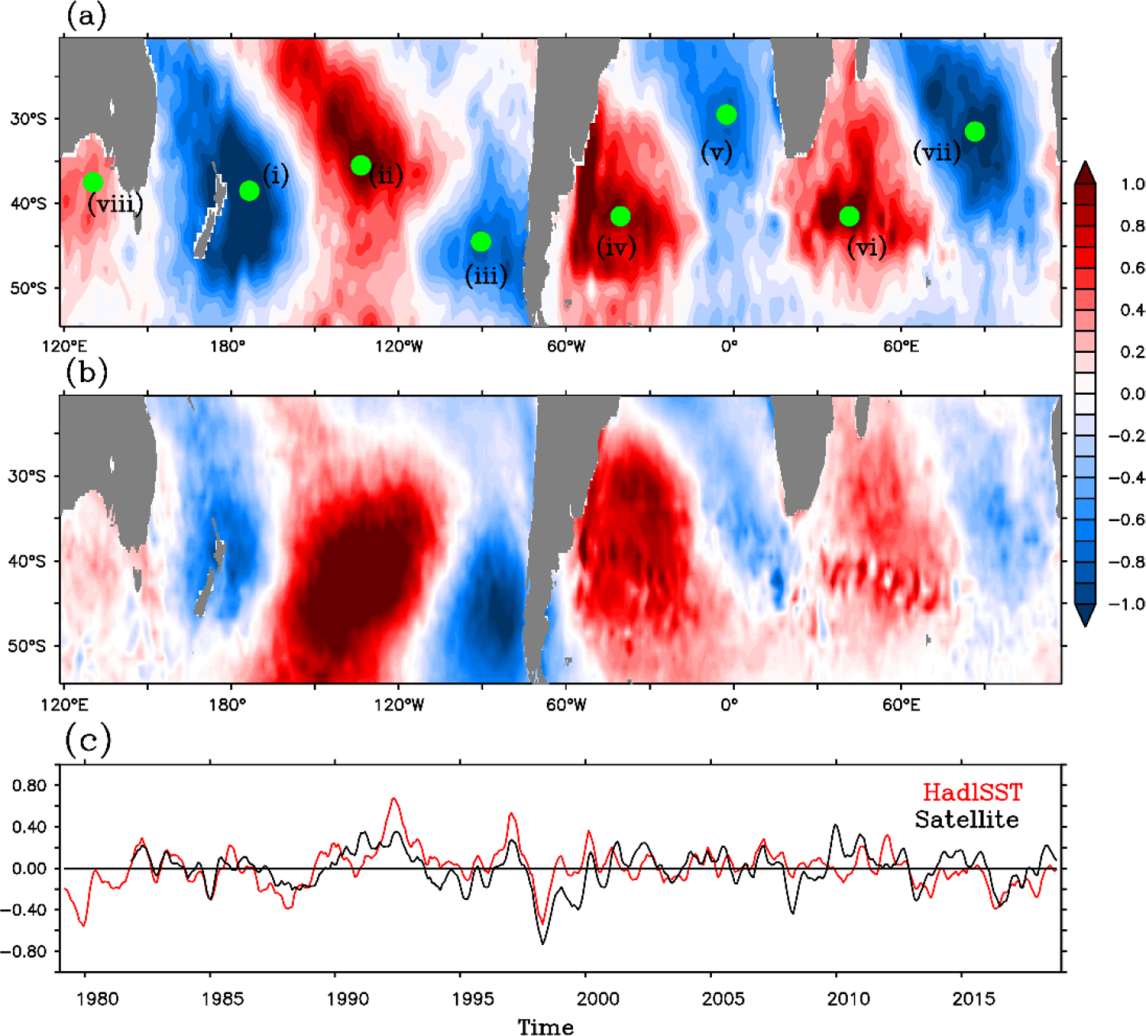
(**a**) Spatial pattern of second leading EOF mode of SST anomaly over the region (20°S-55°S) from HadISST. Green dots mark the points used for correlation maps in Fig. 2. (**b**) Same as (**a**), but for the satellite SST. (**c**) Temporal pattern of second leading EOF mode of SST anomaly, solid red line (for HadISST) and solid black line (for satellite SST). The time series are smoothed with 5 month running mean.

The monthly SST anomalies from Hadley Centre (HadISST) are detrended and decomposed using the empirical orthogonal function (EOF) analysis. In addition, gridded satellite observed SST (henceforth satellite SST) anomalies reported in Merchant et al.^28^ are also used in the analyses to verify the robustness of the results. The first EOF mode of the detrended monthly SST anomalies shows an ENSO like pattern in the Southern subtropics (20°S–55°S) and explains 14.63% (9.41%) of the total variance in HadISST (satellite SST) (Fig. not shown). The time series associated with the first mode (i.e. PC-1) shows a good correlation (correlation coefficient = 0.72) with the Oceanic Niño Index (ONI) at one month lag. The W4 pattern emerges as the second EOF mode of these SST anomalies (Fig. 1a,b) and explains 8.11% and 5.87% of the total variance of HadISST and satellite SST anomalies respectively. This mode is well separated from the first mode and others, North Criteria^29^, which confirms the statistically independent nature of the second EOF mode (Table 1).

**Table 1:** Significance of EOF methods using North Criteria.

HadlSST				Satellite data			
EOF modes	Variance	Error	Significance	EOF modes	Variance	Error	Significance
1st	14.6326	0.94453	Significant	1st	9.41529	0.62908	Significant
2nd	8.11284	0.52368	Significant	2nd	5.87144	0.39230	Significant

The time series of EOF mode-2 of HadISST is considered as the index for W4 (IW4). During positive phase of W4, four positive (negative) SST loading centers are located in the Southern-central Pacific, South-western Atlantic, South-western Indian Ocean, South of Australia (South-eastern Pacific, South-eastern Atlantic, South-eastern Indian Ocean, South-western Pacific Ocean). And, during negative phase polarity of these centers reverses. Compared to other sectors, the signal over the south of Australia seems to be weak. The patterns are similar in satellite SST anomalies and the correlation between both principal components is 0.65. Hereafter analysis in this paper uses the HadISST only.

**Figure 2:**
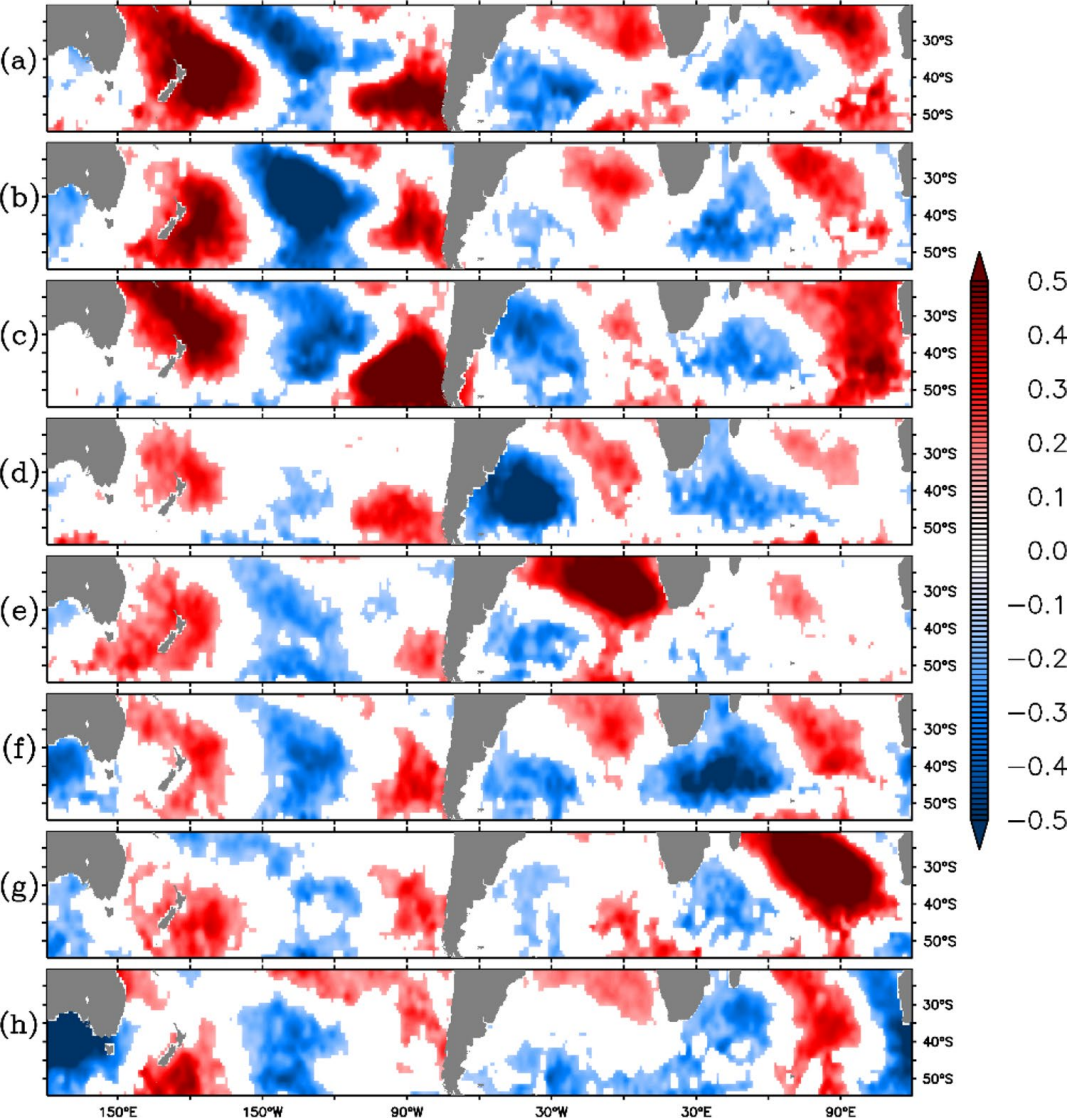
Correlation maps for the time series of points marked in green in Fig. 1 with reconstructed SST anomaly (after removal of first EOF mode related SST anomaly). (**a**)–(**h**) panels refer to points (i)–(viii) respectively presented in Fig. 1. Panel (**b**), (**d**), (**f**), and (**h**) are inverted to keep the same signs of correlation in the same location for all the plots. Values satisfying 99% confidence interval are shaded.

In order to examine the synchronization of the W4 pattern among all the basins, point correlation analysis has been performed. For this purpose, eight points [i(37.5°S, 173.5°W), ii(37.5°S, 133.5°W), iii(44.5°S, 90.5°W), iv(39.5°S, 40.5°W), v(29.5°S, 2.5°W), vi(41.5°S, 41.5°E), vii(30.5°S, 86.5°E), viii(35.5°S, 130.5°E)] corresponding to the loading centres are selected (marked by green dots, i-viii, in Fig. 1a). The time series of SST anomaly is computed at each grid point after removing the contributions of the first EOF mode (henceforth, reconstructed SST anomaly). Further, point correlation is performed for the time series at the loading centers (Fig. 1a) with the reconstructed SST anomaly (Fig. 2a–h corresponding respectively to points (i) to (viii) of Fig. 1a). Only correlation values above 99% significance are shown in the figure. The results suggest a clear existence of wave number-4 pattern across the globe and sign of correlation changes with polarity except for the pole south of Australia (viii in Fig. 1), which apparently is not very much synchronized with the anomalies on both sides of South America (Fig. 2h). But, the south of Australia pole is well correlated with polarities in the Indian Ocean and the Pacific.

After confirming the physical presence of W4 mode in the SST anomalies, we checked its possible relationship with other climatic modes. The W4 lagged cross-correlations with other climate indices are listed in Table 2. SAM and IOD correlations are insignificant with IW4 at 90% confidence interval, whereas ONI, IOSD, SASD show a significant but rather weak correlation (99% confidence) with IW4 at 8, 1 and 0 month lag respectively. Since the subtropical dipoles appear as a wavenumber-3 SST pattern globally^26^, the basin wise unfavorable overlapping of the W4 pattern in Southern Indo-Atlantic sector might prompt lower correlation with SASD and IOSD. Even, the spatial maps of SST anomaly associated with IOSD and SASD do not show the W4 pattern (figure not shown) matching with the results of Fauchereau^16^. Hence, the resulted lower cross-correlations (Table 2) suggest that W4 mode could be to a large extent independent (or not directly related) to other ocean–atmosphere climate phenomena. The correlation with ENSO is also very weak and hence not discussed here. Also, like other climate phenomena, W4 mode is found to be seasonally phase-locked. The IW4 variability is above one standard deviation from December through April (Table 3) suggesting the major appearance of W4 from austral summer to mid-autumn season.

**Table 2:** Cross-correlation between IW4 and climate indices. Monthly lag values are shown in the bracket (positive (negative) lag corresponds to the leading (lagging) of IW4 with the climate indices).

	ONI	SAM	PDO	IOD	IOSD	SASD
IW4	0.2 (+ 8)	− 0.07 (0)	− 0.19 (− 11)	0.08 (12)	0.24 (+ 1)	0.28 (0)

**Table 3:** Monthly Standard deviation values of IW4 (normalized by one Standard deviation).

Jan	Feb	Mar	Apr	May	Jun	Jul	Aug	Sep	Oct	Nov	Dec
1.22	1.4	1.16	1.07	0.93	0.85	0.67	0.68	0.63	0.72	0.91	1.31

### Seasonal evolution of W4

**Figure 3:**
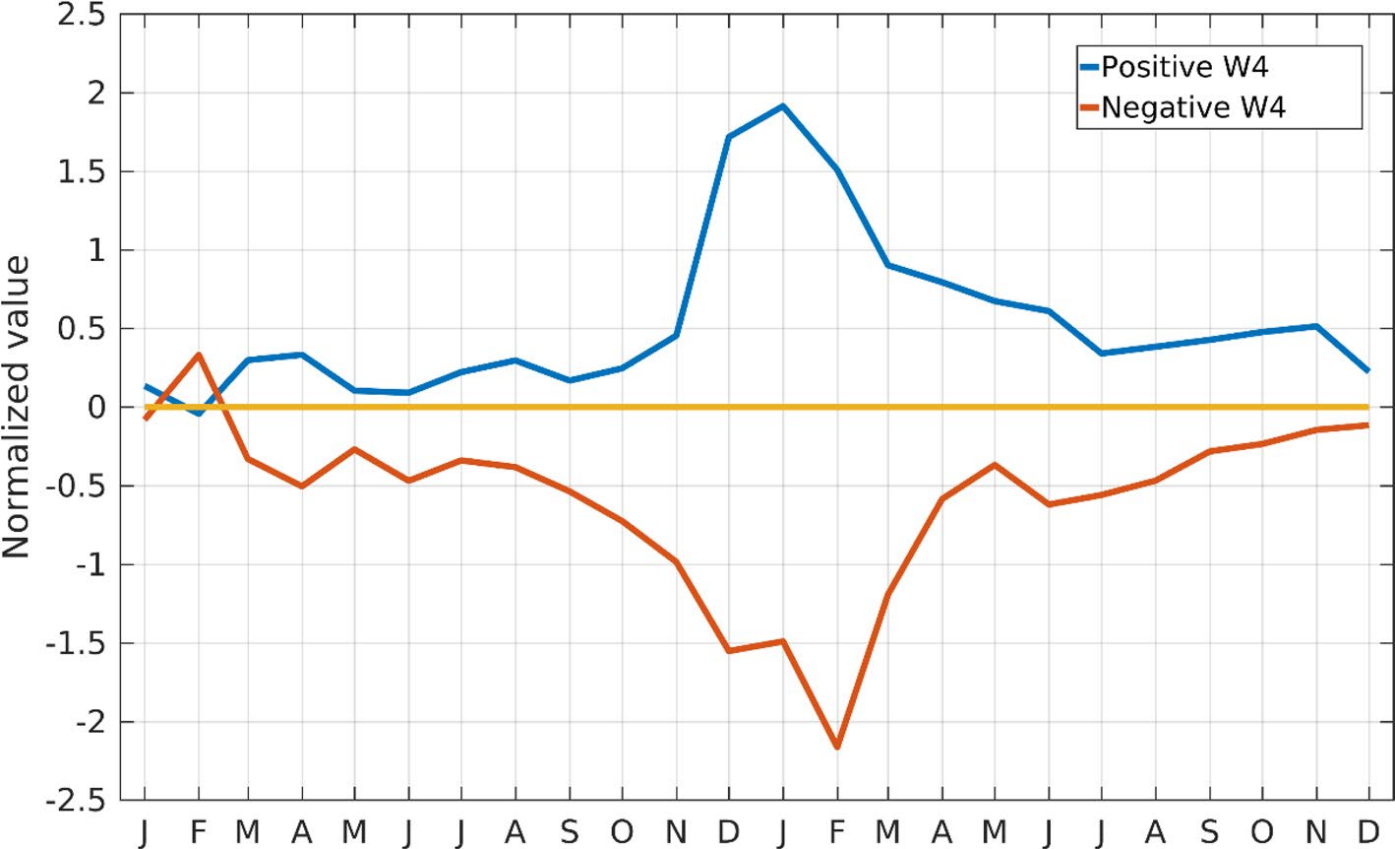
Evolution of the W4 pattern from January of the developing year to the December of the event year. Blue (red) line corresponds to positive (negative) extreme years.

The seasonal evolution of the W4 pattern is studied by analyzing extreme positive and negative event years. Those are identified by a threshold of one standard deviation in the normalized IW4 during austral summer. We found eight positive years (1989–1990, 1991–1992, 1996–1997, 1999–2000, 2004–2005, 2006–2007, 2010–2011 and, 2011–2012) and six negative years (1979–1980, 1984–1985, 1986–1987, 1987–1988, 1997–1998 and, 2017–2018). Composite of the normalized IW4 for extreme positive and negative years for 24 calendar months are shown in Fig. 3. The normalization is carried out by dividing the standard deviation of IW4 with the anomaly of the time series. Care has been taken in selecting the calendar months, such that peak season of occurrence remains at the center of the series. It can be clearly observed that both positive and negative extremes (greater than one standard deviation) occur in the austral summer and/or early autumn. It is interesting that one-month difference is observed in the peaks of positive and negative extremes. The peak for positive extremes occurs in January whereas the peak for negative extremes occurs in February.

**Figure 4:**
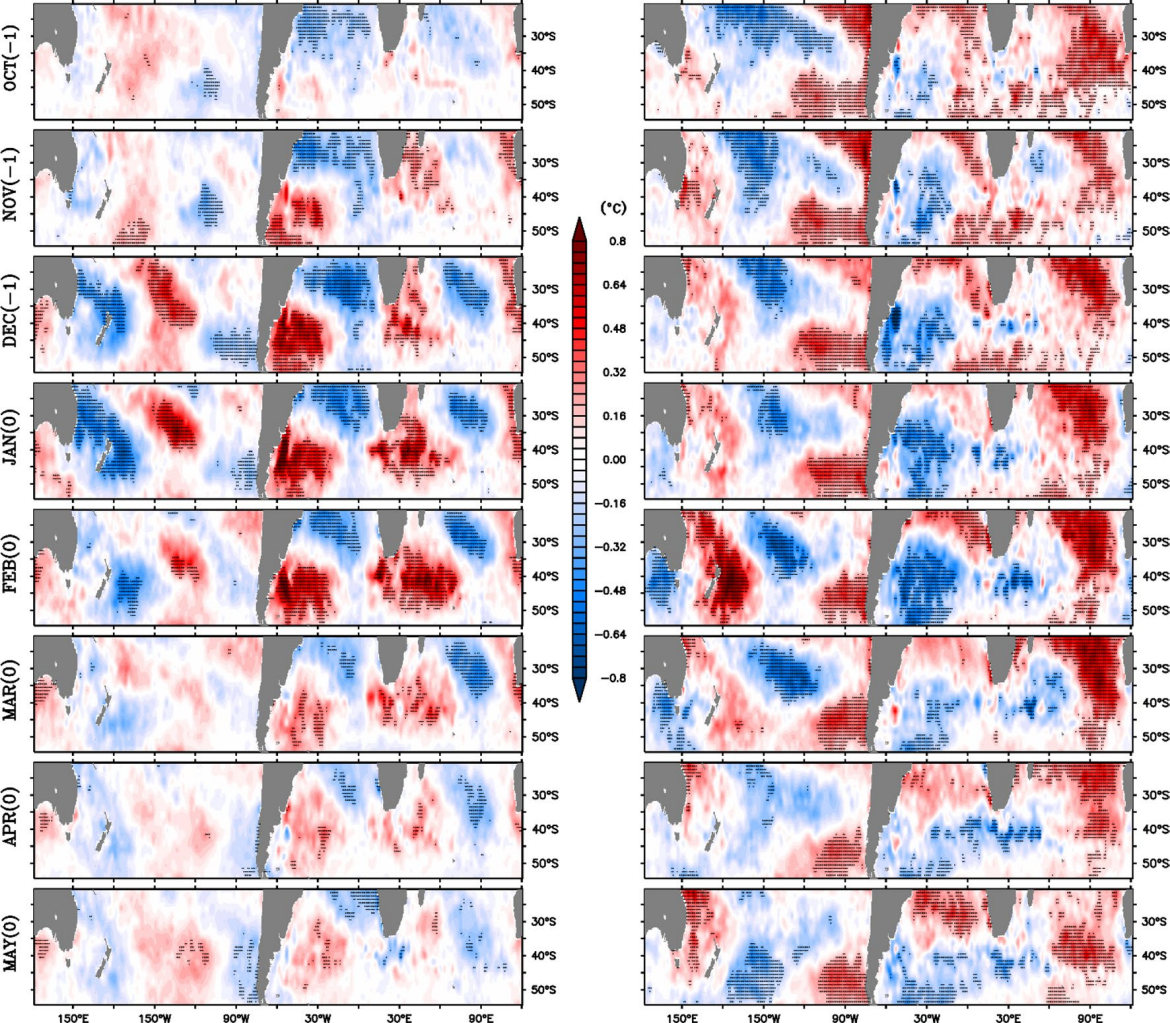
Left (right) panels show the monthly composite maps of positive (negative) W4 years from October (− 1) to May (0) in SST anomaly (in °C). (− 1) suggests the preceding year of the event year (0). Hatched areas represent the values satisfying 95% confidence with a two-tailed t-test. In this analysis HadISST data are used.

To verify further and to examine the evolution of the SST anomaly, a composite for positive and negative years, as identified above, are made from October of the preceding year to May of the event year (Fig. 4). It can be clearly seen that positive (negative) W4 signal is prominent during December, January, and February (December, January, February, and March), and gradually decays in subsequent months (Fig. 4). This further suggests that W4 signal appears in the austral summer, which later persists up to mid-autumn in case of negative years. However, in negative W4 years, the signal seems to be prominent in the region to the south of Australia in February as compared to other seven boxes (Fig. 1a); those boxes that show prominent loading in SST during December (right panel; Fig. 4). This lag in the development of the W4 signal in the region to the south of Australia might be the reason for the weak signal found in the linear EOF analysis (Fig. 1a,b) there.

**Figure 5:**
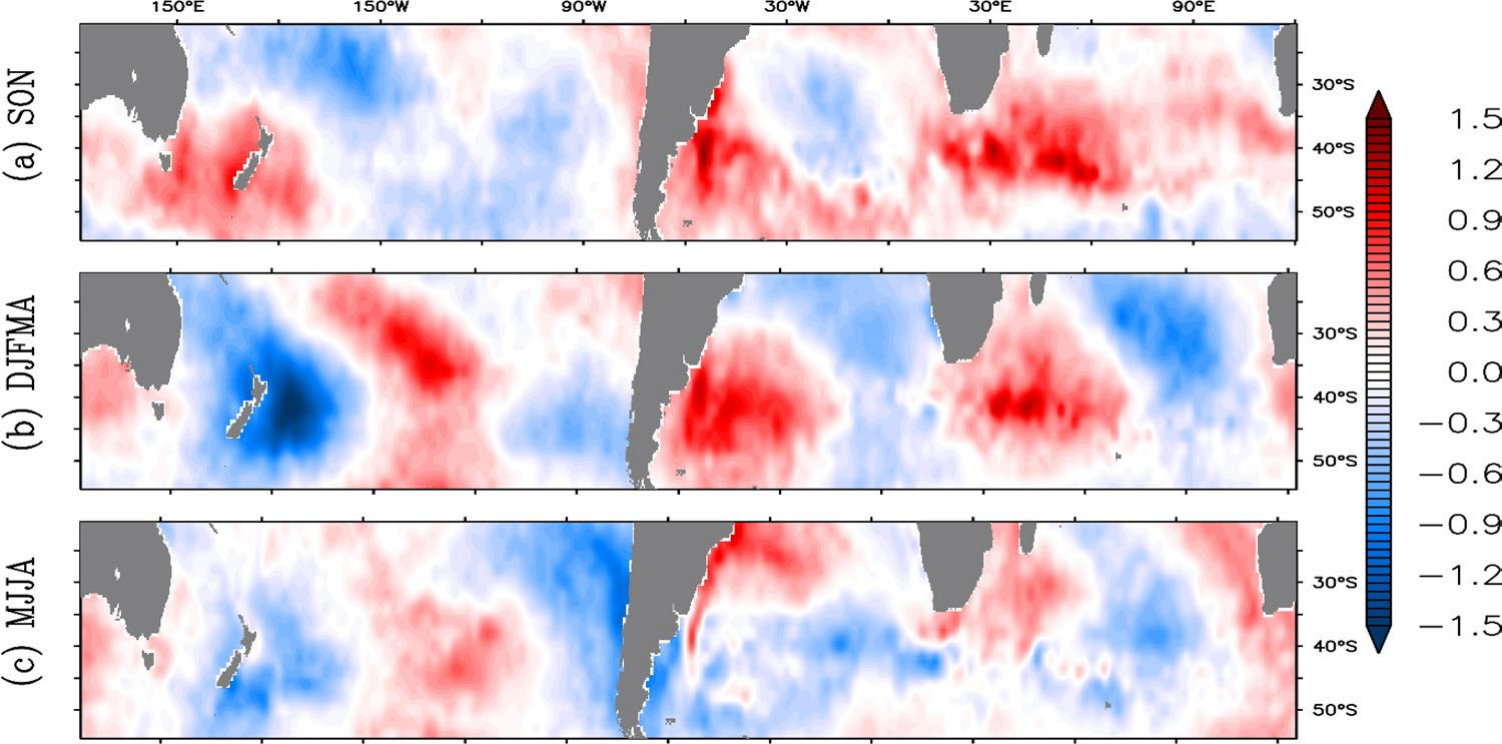
Second spatial mode of the seasonal EOFs (standardized by their respective standard deviations in PCA’s) using SST anomaly over the region (20°S–55°S) from HadISST.

To cross-validate and examine the seasonally evolving pattern, EOF analysis is conducted on the detrended SST anomaly for different seasons over the same region (20°S–55°S). Previous analysis shows that the W4 pattern appears in December through April. So, for our EOF analysis we have divided the whole year into three periods; (1) austral summer through mid-autumn (December–April, DJFMA), (2) late autumn and winter (May–August, MJJA) and (3) spring (September–November, SON). Figure 5 shows the spatial patterns of standardized EOF coefficients for the above three different periods. The standardization for each period is performed by dividing the EOF coefficients by corresponding standard deviation in PCA’s at each grid point. This normalization brings the coefficients to a common range of values for easy comparison. It is interesting to note that, the W4 pattern appears in the second mode for the austral summer and mid-autumn season (DJFMA period). The seasonal EOF suggests the decay of the SST pattern in austral winter over the Indo-Atlantic region despite the presence of a weak signal over the Pacific Ocean. The decay of the pattern is especially observed in the Atlantic region where southwest-northeast orientation pattern turns to northwest-southeast. In other words, the northward (southward) movement of the anomalous SST over the western (eastern) sector of the Southern Atlantic Ocean occurs during austral winter. Similarly, the southern Indian Ocean also witnesses the decay with the development of a tri-polar structure in austral winter. Nevertheless, these results of seasonal EOF agree well with that of monthly composite analysis and confirm the seasonal phase-locking behaviour of the W4 pattern to the austral summer season.

### Possible mechanism

**Figure 6:**
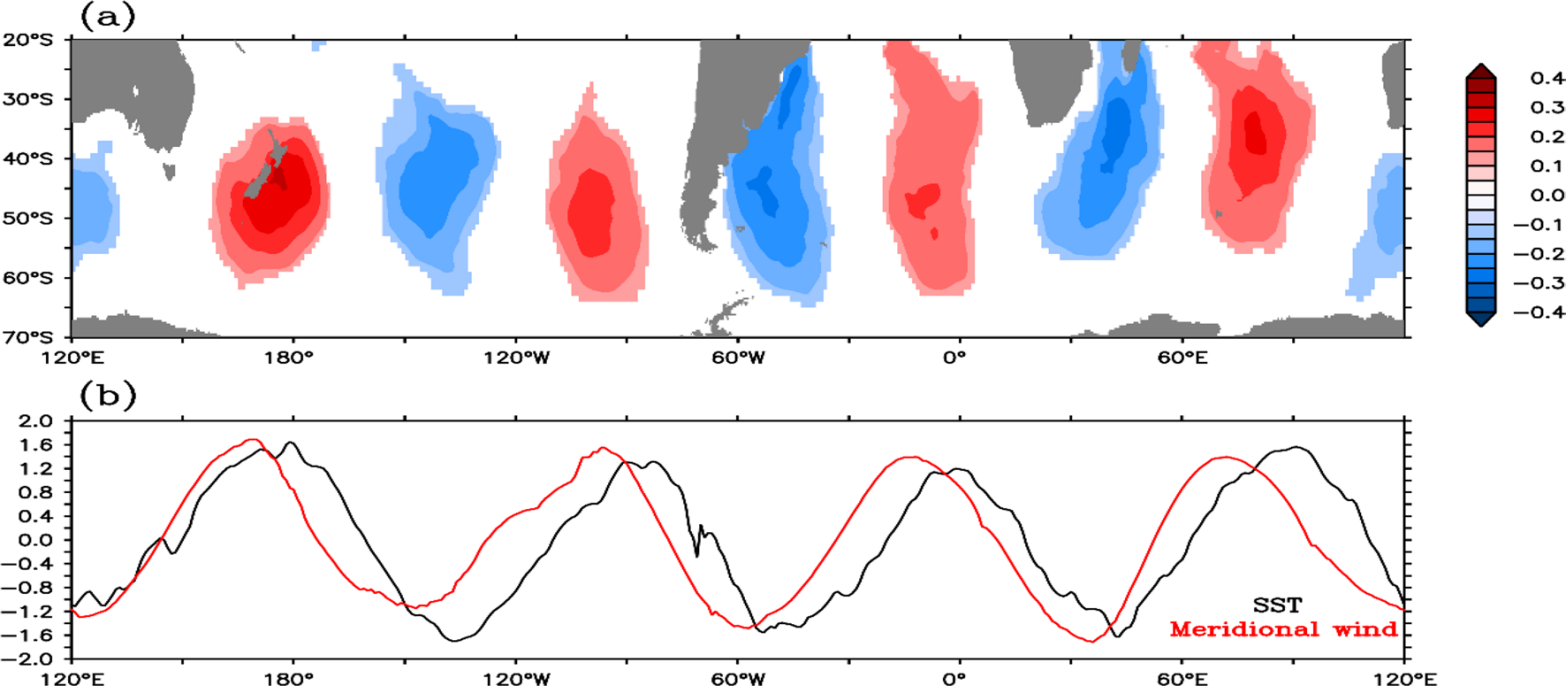
(**a**) Correlation field of above 99% significant between IW4 and meridional wind anomaly at 850 hPa. (**b**) Meridional average of SST (black line; − 1 is multiplied to avoid opposite relation) and meridional wind (red line) from 20 to 55°S.

**Figure 7:**
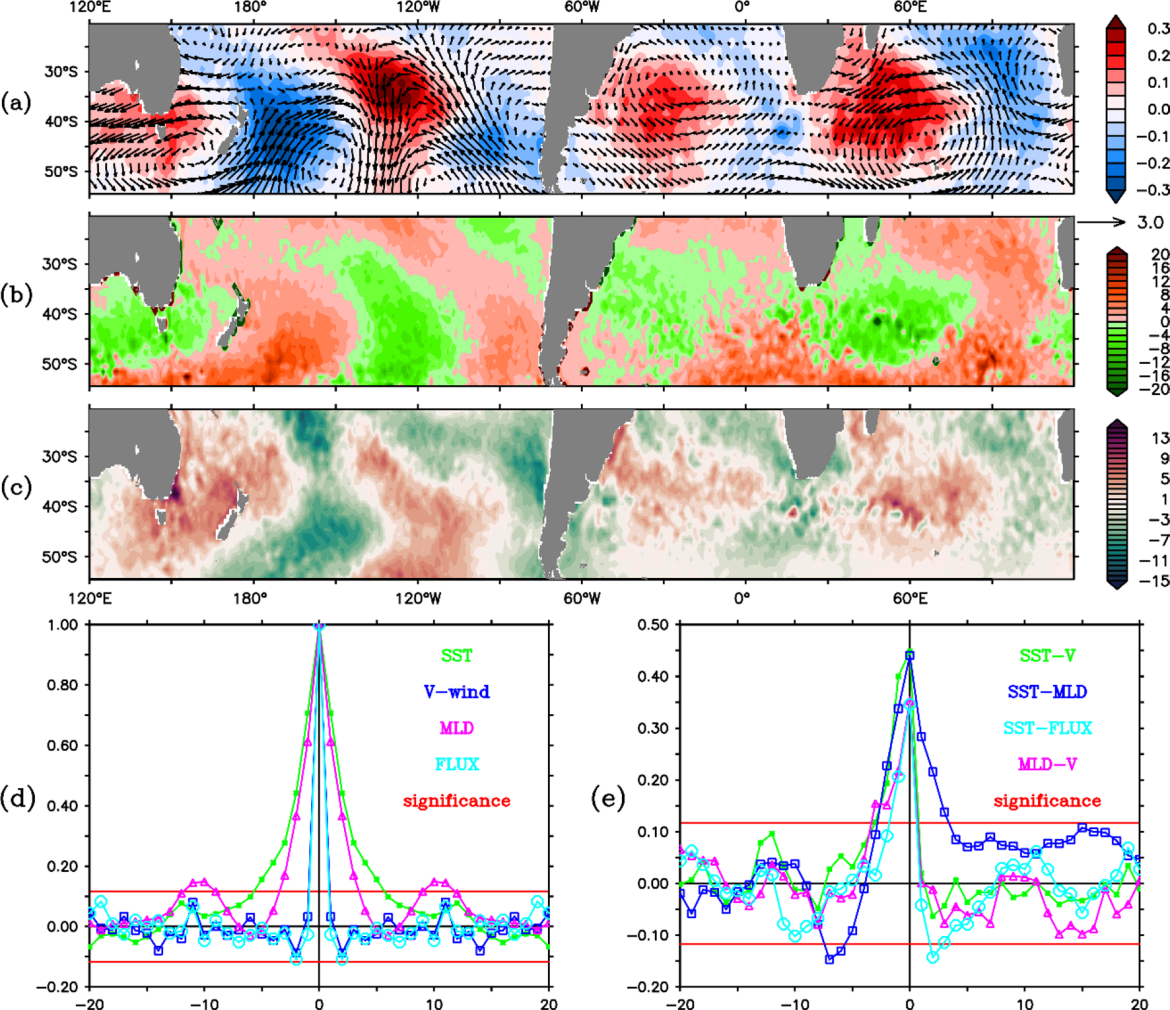
Second SVD mode of anomalous (**a**) SST (shaded) and wind at 850 hPa (vector), (**b**) MLD and (**c**) latent heat flux. (**d**) Auto-correlation and (**e**) cross-correlation of SST, meridional wind (V), MLD and, latent heat flux (FLUX, positive downward) in pairs as indicted by the levels. The x-axis in (**d**) and (**e**) represents the lead/lag in months and y-axis represents the correlation coefficients. Positive lag means first variable is leading the second. Solid red line shows the 99% significance level.

The correlation between meridional wind (V) at 850 hPa and IW4 indicates the co-existence of the signal in the ocean–atmosphere system (Fig. 6a). However, there is a phase difference of approximately 10° between corresponding loading centers (Fig. 6b). Hence, to explore the interaction between them, maximum covariance analysis (MCA) is performed among several oceanic and atmospheric parameters. Similar to previous results that appeared in the EOF analysis, W4 mode is captured in the second MCA mode between SST, meridional wind (V), mixed layer depth (MLD), and latent heat flux (Fig. 7a–c). A correlation of 0.82 is found between the W4 mode-2 SST time series computed using EOF and MCA methods. It confirms the robustness of W4 signal as it appears in both the methods. Also, unlike the slight phase shift in SST and V discussed earlier, MLD and latent heat flux patterns match well with the SST W4 pattern. The difference in centre of actions in SST (Figs. 1, 2, 4) and meridional wind (Fig. 6) may be explained through their interactions with latent heat flux and MLD (Fig. 7b,c). The favourable region for release of latent heat flux is based upon air-sea humidity gradient and divergent wind (figure not shown). Such regions are different from that of high meridional wind variability. Heat loss/gain in the ocean due to anomalous latent heat flux (about 10–11 W m^−2^; Fig. 7c) is almost five times that of anomalous sensible heat flux (about 2–3 W m^−2^; Fig. S1b), revealing the dominancy of latent heat flux in coupling of air-sea interaction. As a result, the net heat flux anomaly (Fig. S1a) displays very similar pattern and magnitude to that of latent heat flux. However, it is to be noted that the sensible heat flux also shows a similar W4 pattern (Fig. S1b) contributing to the total heat flux (Fig. S1a). The loading centre of the sensible heat flux matches with that of the maximum meridional wind variation. The link could be explained through the anomalous equator(pole)ward wind that may bring cold (warm) air to the subtropical region. This contributes to the air-sea temperature gradient and results in variation in the sensible heat flux.

The auto- and cross-correlations of the variables as shown in Fig. 7d,e show the evolution of W4 mode over its development cycle. In agreement with previous discussions, SST autocorrelation shows its five-month evolution period through the persistence. However, the MLD persists for three months and latent heat flux persists for only 1 month. From the cross-correlation of SST-MLD, MLD-V, and SST-V, we can conclude that the SST and MLD signals are generated in response to the wind over two months of preconditioning period. Also, latent heat flux is generated along with the meridional wind (correlation = 0.71). Conversely, it is interesting to see that, when SST leads, all the atmospheric variables disappear (Fig. 7e). However, MLD persists significantly up to 3 months after the SST W4 pattern peak and helps the W4 to persist over the region for a while. Another fascinating result is the reversal of the latent heat flux at that time, after leading SST pattern for two months (Fig. 7e). All these indicate that the SST W4 mode starts to force the atmosphere after its establishment, which results in the reversal of the flux pattern and their cross-correlations. The air-sea interaction can be explained in the composite of latent heat flux (Fig. S2). During positive years, gain/loss in latent heat flux (left panel; Fig. S2) of about 12 Wm^−2^ in November and December is capable of generating SST anomaly up to 0.6ºC (left panel; Fig. 4) with a constant MLD of 50 m in absence of other processes^3^. SST reaches its peak in January (Fig. 3), and starts forcing the atmosphere upon which a transition phase is observed during January and February. In agreement with the MCA analysis the latent heat flux flips its sign in March and April, which is 2 month of post-maturity in SST anomaly. The scenario is opposite in accordance with different peak time in SST anomaly (Fig. 3) during negative years (right panel; Fig. S2). A similar process is described elsewhere^30^. Accumulating all the above results, the generation mechanism can be summarized as follows.

To the west (east) of an anomalous anticyclone, divergent wind, associated latent heat flux, and detrainment (entrainment) warming (cooling) are enhanced. The wind-induced SST warming (cooling) would favour in decreasing (increasing) the mixed layer depth behind (ahead of) the anticyclone^30^. Variation in MLD (Fig. 7b) and its longer tenacity (Fig. 7d) indicate that the shallower (deeper) MLD support the surface warming (cooling) as incoming solar radiation is distributed in a thinner (thicker) layer^31^. Oppositely, SST warming (cooling) on the western (eastern) side favours strengthening of the anticyclone by helping the source, not known yet, of the anomalous wind. This positive feedback mechanism helps to build up the pattern to its peak, after which the pattern starts to force the atmosphere. As a response to this decoupling, the atmospheric signal dies very quickly (Fig. 7e). Also, due to the breakdown of positive thermodynamic feedback loop of anomalous wind and upper ocean dynamics, W4 pattern starts decaying slowly following the MLD. Since the Southern subtropics has seasonally distinct weaker wind and strong insolation during austral summer^26^, MLD anomalies continue to persist sustaining the SST pattern over the region for a while. Nevertheless, SST pattern persists up to March–April of the event year due to the long memory of the ocean. The opposite scenario happens in case of anomalous cyclonic circulation, which leads to a negative W4 pattern.

### Australian rainfall variability linked to W4 pattern

**Figure 8:**
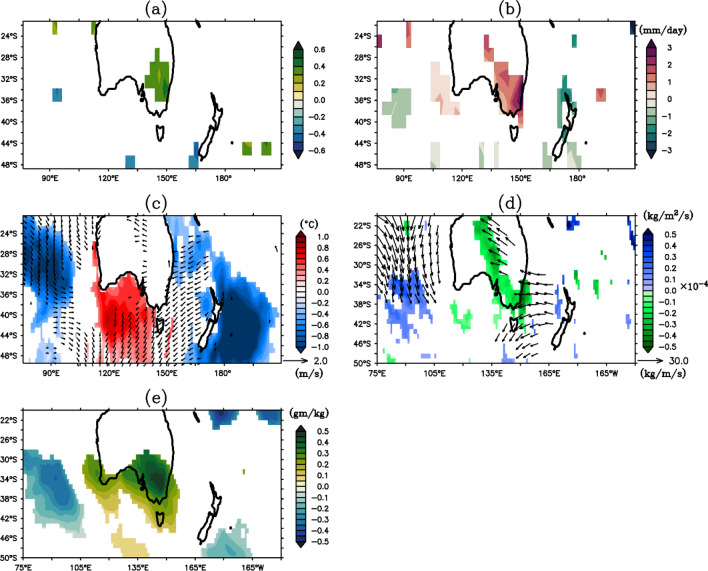
(**a**) Correlation field between IW4 and rainfall anomaly during austral summer. Composite of positive minus negative W4 extreme years in February (**b**) rainfall (in mm/day), (**c**) SST(shaded; in °C) and divergent wind at 850 hPa (vector; in m/s), (**d**) vertically integrated moisture divergence (shaded; in kg/m^2^/s) and moisture transport (vector; in kg/m/s) for whole atmosphere (i.e. surface to 1 Pa), and (**e**) vertically integrated specific humidity (in gm/kg) for whole atmosphere (i.e. surface to 1 Pa). Values less than 95% significance level are suppressed.

Inter-annual variability of Australian rainfall is strongly related to the anomalous SST over the surrounding Oceans and large-scale atmospheric circulations in relation to different tropical and extra-tropical climate modes^32–37^. Especially, south-eastern Australian (SEA) rainfall is dominantly influenced by IOD^34^. Besides, the rainfall over the SEA has experienced challenging climate variability in the last two decades^35^. Further, correlation analysis shows that about 40–50% variability in precipitation over SEA is linked to W4 during austral summer (Fig. 8a). To further peruse and understand their connection, a composite of difference in precipitation during extreme positive and negative W4 years is presented (Fig. 8b). The choice of composite is based on the relationship between positive and negative years that are generally opposite in phase through the seasonal evolution cycle (Fig. 4). It can be clearly seen that the SEA gets heavy rainfall (1.5–2.0 mm/day) during positive W4 events in February (Fig. 8b) as compared to other regions. Additionally, SST and several atmospheric variables (divergent of wind at 850 hPa; vertically integrated moisture divergence and moisture transport for total atmosphere) are consistent with the rainfall composite during February (Fig. 8c–e) at a 95% confidence interval. Cold SST anomalies develop over the southwestern Pacific and the southeastern Indian Ocean, crammed with the warm SST anomaly to the South of Australia (Fig. 8c), during positive W4 events. In response, anomalous winds diverge from the colder region to the warmer oceanic region. This situation favors anomalous easterlies (westerlies) on the eastern (western) side of the continent (Fig. 8c). Along with the wind, moistures are transported to the Australian landmass (Fig. 8d,e). Moreover, the increase in specific humidity over the same region (Fig. 8e) supports the enhanced rainfall over SEA (Fig. 8b). Conversely, during negative W4 years, the presence of warm SST anomaly over the western and eastern side of Australia packed with cold SST anomaly in the region to the south of Australia reverses the atmospheric circulation. Consequently, the wind and moisture diverge reducing the tropospheric specific humidity that lead to decreased rainfall over SEA.

## Discussion and summary

Two high latitude modes, the Antarctic circumpolar wave^15^, and the Antarctic Oscillation^38^ have been noted in the Southern Hemisphere. Further, Wang^26^ showed the presence of a global zonal wave number-3 in southern subtropical SST during austral summer-autumn. Besides, the presence of subtropical dipoles in the southern subtropical ocean basins have been reported in regional scale^3,10,39^. Examining the co-variability in SST dipoles between the southern- Indian and Atlantic Oceans, Fauchereau^16^, reported the presence of a W4 pattern in the atmospheric anomalies during austral summer. This pattern in fact was already observed in brightness temperature over the Southern subtropics^24^. However, its global consistency, spatial phase change over time (called mode) and temporal phase-locking behaviour have not been studied and discussed yet. In this study, we found that there exists a W4 mode in the SST anomalies over the southern subtropics during austral summer through to mid-autumn season. A higher variance is noted in the W4 mode (especially in the Indian Ocean) derived from the HadlSST data, as compared to satellite data, perhaps related to its EOF based reconstruction. Sub-tropical central Pacific Ocean was observed to serve as a major SST anomaly region among all the loading centers in the W4 pattern. Because of thermodynamic coupling between the atmosphere and upper ocean, the W4 pattern emerges and grows until it forces the atmosphere. Atmospheric response to the W4 SST breaks the feedback loop of anomalous wind and upper ocean dynamics resulting in the decay of the pattern. Nevertheless, it persists up to March–April of the event year due to the memory in the ocean. However, the source of anomalous atmospheric circulation at the beginning of W4 pattern  is out of the scope of this study and kept open for future works. On the other side, the SST anomaly due to the W4 event forces the atmosphere to cause a substantial rainfall variation over southeastern Australia. This notable connection between W4 SST mode and Australian rainfall develops a better understanding of the dynamics of the continental rainfall especially to the SEA region of Australia.

In summary, a global wave number-4 pattern in SST anomaly has been reported through EOF analysis in the southern mid-latitude. Using North Criteria^29^ and point correlation analysis, the significance of the EOF mode, and global consistency of the pattern have been verified. Correlation analysis suggests, this SST pattern is not dependent on other known climate phenomena that develop in tropical and southern extra-tropical regions. This W4 SST mode shows a seasonal phase-locking behaviour to the austral summer with an extension of persistence up to early/mid-autumn. Atmosphere–ocean coupling helps in generating the W4 pattern, which later dies down due to the break-down in that positive feedback loop of the upper ocean and atmosphere. During positive phase of the W4 event, the cold SST anomaly over the south-eastern and -western side of Australia creates an anomalous divergence circulation. This favours the moisture transport towards the SE region of the continent. As a consequence, the specific humidity increases and causes an above normal rainfall in a SE-NW axis over Australia. This scenario reverses in case of a negative W4 event.

We hope that this distinct W4 pattern in SST over the southern subtropics will help in understanding the ocean dynamics over the region. The exploitation of its (W4) relationship with SEA summer rainfall may lead to improve the predictability of rainfall over that region. However, a coupled model study extended in scope is needed to comprehend the full repercussion of the SST mode.

## Material and methods

In the present study, we use monthly mean SST and MLD data from Hadley Centre Global Sea Ice and Sea Surface Temperature (HadISST^40^) and ORAS-5 reanalysis^41^ having a spatial resolution of 1° × 1° for the period 1979 to 2018 respectively. For validation of EOF analysis, available gridded satellite data^28,42^ of same spatial resolution spanning from September, 1981 to December, 2018 is used. Atmospheric variables (horizontal wind at 850 hPa, sensible and latent heat fluxs, vertically integrated moisture divergence, and specific humidity) of similar spatio-temporal resolution to that of HadlSST are adopted from ERA-5 reanalysis products^43^. Here, the authors also used the monthly mean rainfall data from the CPC Merged Analysis of Precipitation^44^ of 2.5° × 2.5° spatial resolution over the same interval and period. Monthly anomaly is obtained by subtracting the monthly climatology from its corresponding monthly value after removing the linear trend using a least-squares fit at each grid point. The study includes the region between 20 and 55°S in the Pacific, the Atlantic and the Indian Oceans of the Southern Hemisphere. To identify the dominant linear modes, a detrended monthly SST anomaly was decomposed using an EOF over the region. Further, North criteria^29^ was used to test the significance and independency of EOF modes. Pearson’s linear correlation coefficient of ‘n − 1’ degrees of freedom has been used to calculate the point correlation map. The correlation coefficient is tested against the null hypothesis using t-test statistic, $t = r \times \surd \left[ {\left( {n - 2} \right) \div \left( {1 - r^{2} } \right)} \right]$; where r-correlation coefficient, n-sample size; which has student-t distribution with ‘n-2’ degrees of freedom. Student two-tailed t-test is used to test the significance in the composite analysis at 95% significance level. For seasonal EOF analysis, first, the detrended monthly SST anomaly data was split up into three segments, (1) austral summer through mid-autumn (DJFMA), (2) late autumn and winter (MJJA) and (3) spring (SON) for our study. Then, EOF analysis is carried out for each of the segment separately over the same region (20°S–55°S). Now, to check the seasonal evolution, the EOF coefficients are divided by corresponding standard deviation in PCA’s at each grid point, which helps to bring the EOF coefficients to a common range.

### Maximum covariance analysis (MCA)

To explore the air-sea interaction mechanism, MCA is used in this study. It is a singular value decomposition (SVD) analysis applied to a cross-covariance matrix of two different variables. In this study, the SST anomaly is kept as a fixed variable while the other variable is chosen from wind/ MLD/ latent heat flux to construct the corresponding cross-covariance matrices. These cross-covariance matrices are further used to perform the SVD analysis. Since the W4 pattern matures during the austral summer season, variables are chosen for DJF months (austral summer) to feed to the MCA analysis. To understand the evolution of the mode-2 patterns, the spatial patterns obtained from MCA analysis (left and right singular vectors correspond to the pattern of first and second variables respectively) are projected on the original anomalous data (which contains all calendar months). Thus, the generated time series contains the variability of mode-2 signals only. To bring the SVD coefficients to a common range, each time series is divided by its standard deviation and, simultaneously multiplied with respective spatial patterns. Then auto-correlation and cross-correlation analysis are performed among the parameters.

### Indices

Climate indices used in the analyses are obtained from different sources; Oceanic Niño Index (ONI; https://origin.cpc.ncep.noaa.gov) from CPC, NOAA using ERSSTv5^45^; Pacific Decadal Oscillation (PDO; https://www.ncdc.noaa.gov) index from NCDC, NOAA using ERSSTv5^45^; Indian Ocean Dipole (IOD) index (https://www.esrl.noaa.gov) from ESRL,NOAA using HadlSST^40^; Southern Annular Mode (SAM) index^46^ (https://climatedataguide.ucar.edu) from NCAR/UCAR. Besides, calculated Indian Ocean Sub-tropical Dipole (IOSD)^3^, and South Atlantic Sub-tropical Dipole (SASD)^31^ index were used to examine their impact on W4.

## Supplementary Information

Supplementary information 1.
