# Supplementary material for: Global wave number-4 pattern in the southern subtropical sea surface temperature
Source: Sci Rep. 2021 Jan 8;11:142. doi: 10.1038/s41598-020-80492-x (PMC7794447; doi:10.1038/s41598-020-80492-x)
Supplement: Supplementary file 1 — Supplementary information 1. [file 41598_2020_80492_MOESM1_ESM.docx]

Supplementary Information

**Global Wave Number-4 Pattern in the Southern subtropical Sea Surface Temperature**

Balaji Senapati^1^, Mihir K. Dash^1*^, and Swadhin K. Behera^2^

^1^Centre for Oceans, Rivers, Atmosphere and Land Sciences, Indian Institute of Technology Kharagpur, Kharagpur, West Bengal, India.

^2^Application Laboratory, Japan Agency for Marine-Earth Science and Technology, Yokosuka, Kanagawa, Japan.

*Corresponding author: Mihir K. Dash ([mihir@coral.iitkgp.ac.in](about:blank))

**Supplementary figures.**


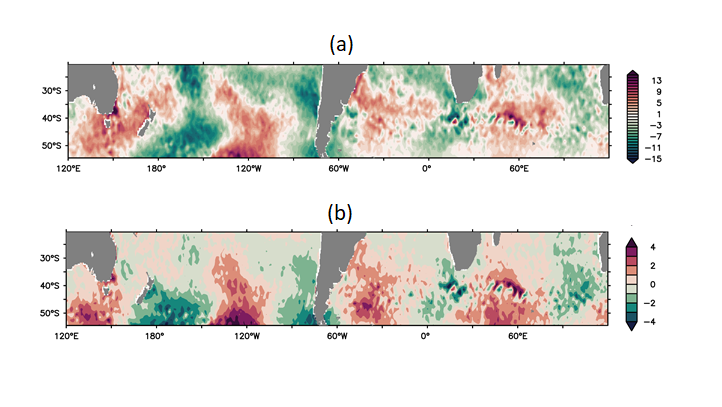
**Figure S1**. Second SVD mode of anomalous (a) total heat flux (in W m^-2^) (b) sensible heat flux (in W m^-2^). Positive values represents downward heat flux.


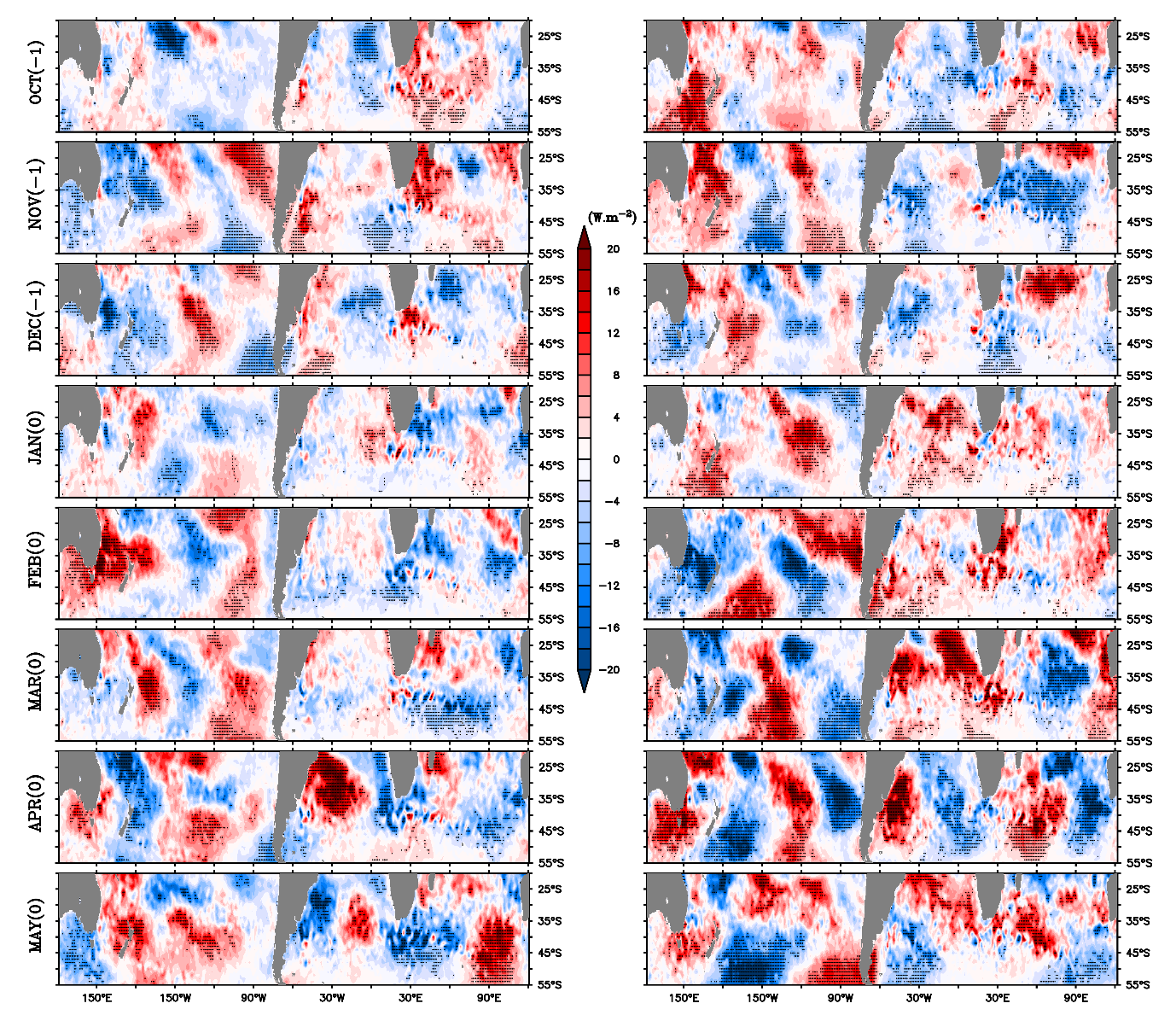


**Figure S2**. Left (right) panels show the monthly composite maps of positive (negative) W4 years from October (-1) to May (0) in SST anomaly (in W m^-2^). (-1) suggests the preceding year of the event year (0). Hatched areas represent the values satisfying 90% confidence with a two tailed t-test.
